# Ferroptosis, a Distinct Form of Cell Death, and Research Progress on Its Modulators

**DOI:** 10.3390/ph18121785

**Published:** 2025-11-24

**Authors:** Junwei Chen, Zhonglang Gou, Guoxin Yang, Lingli Zhou, An Na Kim, Wenchao Shi, You Zhou

**Affiliations:** 1 State Key Laboratory of Resource Insects, College of Sericulture, Textile and Biomass Sciences, Southwest University, Chongqing 400715, China; (L.Z.); 2 CDD Engine, Lino Lakes, MN 55014, USA

**Keywords:** ferroptosis, iron, small molecule, modulator, cancer, neurological disorders, drug discovery

## Abstract

Ferroptosis, an iron-dependent and lipid peroxidation-driven mode of regulated cell death, holds significant pathological significance. Its dysregulation manifests in dual facets: inhibition promotes tumorigenesis, whereas overactivation aggravates neurological disorders and organ injury. This paper systematically reviews the core molecular mechanisms of ferroptosis and provides a comprehensive summary of recent advances in its modulators: inducers classified by targets (GPX4 axis, iron metabolism, lipid metabolism, and GPX4-independent antioxidant pathways) and inhibitors classified by source (synthetic and natural). It places a particular focus on summarizing and analyzing the optimization strategies, mechanisms of action, existing limitations, and future directions for novel ferroptosis modulators, to offer valuable insights for future drug development targeting ferroptosis.

## 1. Introduction

Ferroptosis, defined by Dixon and colleagues [1] in 2012, is a distinct form of cell death that is initiated by intracellular free ferrous iron (Fe^2+^) overload and excessive accumulation of lipid peroxides (LPO), causing damage to the cell membrane and organelle membranes. In contrast to traditional forms of regulated cell death—such as apoptosis, autophagy, necroptosis, and pyroptosis—ferroptosis is characterized by distinct morphological features. These include cellular rounding and specific mitochondrial alterations, including shrinkage, fewer cristae, and increased membrane density, while the nucleus remains intact [1,2]. Biochemically, ferroptosis manifests as iron accumulation and lipid peroxidation. The accumulation of intracellular Fe^2+^ directly generates excessive reactive oxygen species (ROS) through the Fenton reaction, triggering lipid peroxidation [2]. Genetically, ferroptosis is associated with dysregulation in the metabolism of iron, lipids, and amino acids, involving abnormal expression of specific genes. The upregulation of acyl-CoA synthetase long-chain family member 4 (ACSL4) and the downregulation of glutathione peroxidase 4 (GPX4) are notable instances (the detailed mechanisms of action of these genes are discussed in Section 2) [3,4].

**Figure 1:**
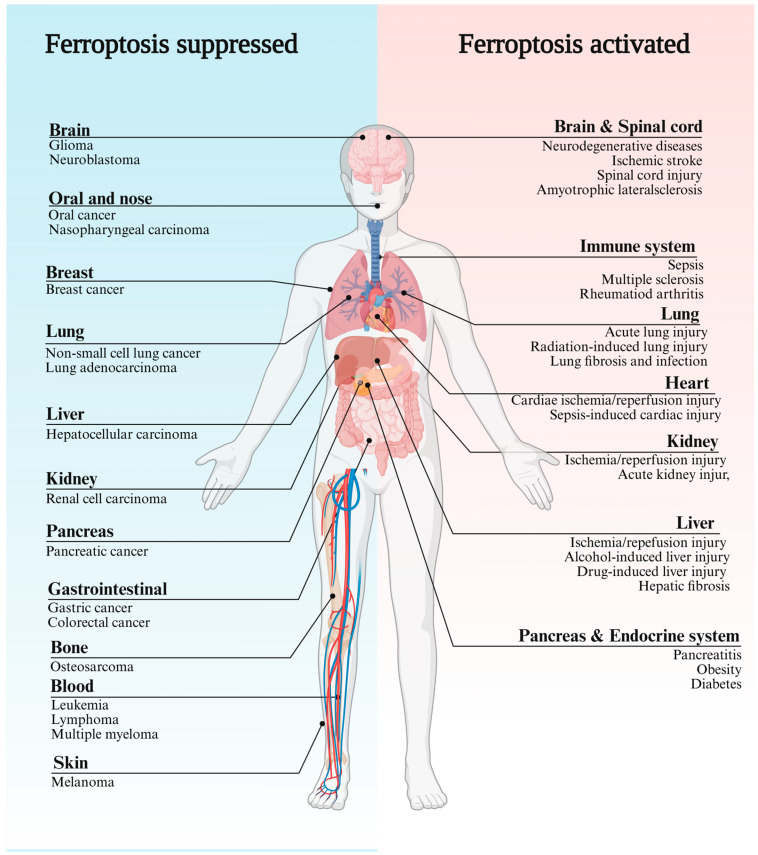
The pathological significance of ferroptosis. This figure systematically illustrates the broad associations between ferroptosis and human diseases. In cancers (left half, blue background), ferroptosis resistance contributes to tumor progression and therapy resistance, whereas inducing ferroptosis has emerged as a potential therapeutic strategy. The right half (red background) represents injury-related disorders associated with hyperactivation of ferroptosis. Understanding the mechanisms and regulation of ferroptosis across diverse diseases may facilitate the development of novel therapeutic interventions targeting this pathway. Created in BioRender. Chen, J. (2025) https://BioRender.com/727asls, accessed on 2 October 2025. This review primarily focuses on advances in ferroptosis inducers and inhibitors; detailed molecular mechanisms in specific diseases are not explored herein. Interested readers could refer to references [5,6,7,8].

As a relatively new form of cell death, ferroptosis has garnered significant attention in the medical field. Growing evidence suggests that ferroptosis plays a role in various human diseases due to dysregulation in both directions. Inhibition of ferroptosis is associated with tumor development and resistance to treatment, while its excessive activation is involved in conditions like neurological disorders (NDs) and organ ischemia/reperfusion (I/R) injury (Figure 1) [5,6,7,8]. Thus, targeting ferroptosis offers a dual therapeutic strategy: triggering ferroptosis in cancer with inducers, while protecting healthy tissues from ferroptosis-related damage using inhibitors. In cancer treatment, RAS-selective lethal small molecule 3 (RSL3), a ferroptosis-specific inducer, demonstrates antitumor efficacy by inducing ferroptosis in colorectal cancer via GPX4 inactivation and ROS production [9]. Sulfasalazine induces ferroptosis in breast cancer cells by inhibiting GPX4, decreasing glutathione (GSH) levels, and upregulating iron-related proteins [10]. In addition to directly inhibiting tumor growth, small-molecule ferroptosis inducers can enhance chemosensitivity. Combining erastin (another ferroptosis-specific inducer) with doxorubicin, temozolomide, or cisplatin shows strong synergistic effects in cancer therapy [11,12]. Conversely, inhibiting ferroptosis to reduce cell death is of great significance in the treatment of traumatic diseases. I/R causes significant cell death and inflammation in organs, exacerbating tissue damage in conditions like stroke and heart/hepatic/and renal dysfunction. Ferroptosis is pivotal in I/R injury, linked to ROS overaccumulation due to ischemia [13]. Consequently, iron chelation therapy and pharmacological inhibition of ferroptosis have both been shown to be effective in alleviating heart failure resulting from acute and chronic I/R injury in mice [14]. In Alzheimer’s disease (AD), neuronal ferroptosis triggered by iron overload is closely linked to the accumulation of misfolded proteins [15]. Research indicates that the iron chelator deferoxamine improves spatial memory in APP/PS1 transgenic AD mice, lowers soluble β-amyloid levels in the cortex and hippocampus, and reduces GSK3β activity, thus mitigating oxidative stress [16].These findings underscore the potential of inhibiting ferroptosis as a promising treatment approach for NDs.

The research progress of ferroptosis modulators, with their dual therapeutic potential in disease treatment, holds significant value for advancing drug development. This paper will analyze the fundamental molecular mechanisms of ferroptosis, followed by a discussion on inducers and inhibitors. Classical compounds, such as erastin, RSL3, and ferrostatin-1 (Fer-1), will be briefly reviewed for their mechanisms and limitations, with a focus on novel modulators identified through high-throughput screening, structural optimization, and natural product exploration. We will delve into their structure–activity relationships (SARs), mechanisms of action, and therapeutic effects in relevant disease models. This review presents a thorough analysis and discussion of the potent small-molecule ferroptosis modulators identified in recent years. The focus of this paper is exclusively on small-molecule compounds. By directly comparing the structures of parent compounds and their derivatives, we discuss key optimization strategies and the identified pharmacophores, while establishing intrinsic connections between chemical structures, biological activity, and mechanisms of action. The aim is to provide strategic insights and a foundation for the development of specific and safer drugs targeting ferroptosis.

## 2. Molecular Mechanisms of Ferroptosis

**Figure 2:**
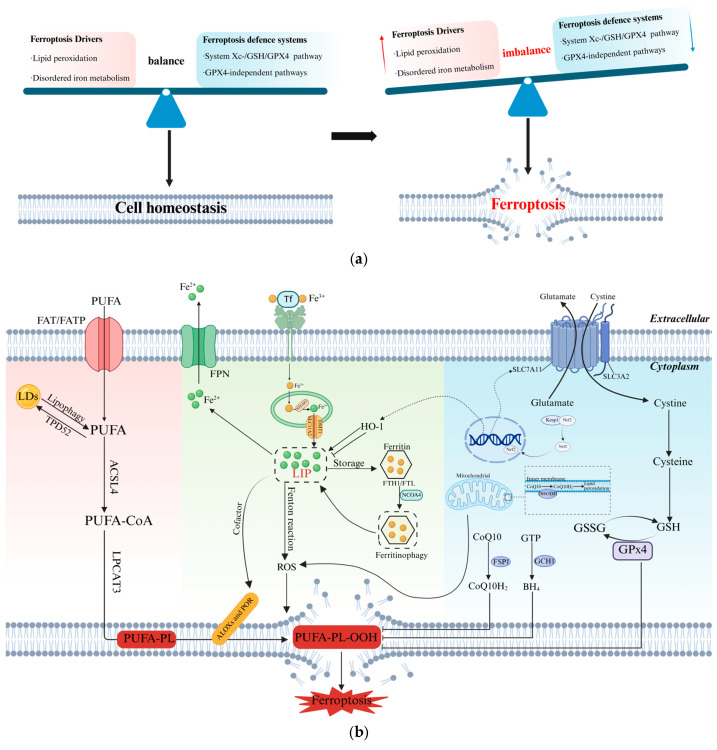
The core molecular mechanism of ferroptosis: (**a**) Schematic representation of ferroptosis as an antagonism between drivers and defense systems. Disruption of this balance triggers ferroptosis. The red upward and blue downward arrows denote promoting and inhibitory effects on ferroptosis, respectively. (**b**) Detailed intracellular signaling pathways regulating ferroptosis, including key molecular components and their functional interactions. Created in BioRender. Chen, J. (2025) https://BioRender.com/jdt977b (accessed on 4 August 2025).

Ferroptosis results from an imbalance in redox homeostasis between its drivers and defense systems [17]. Disordered iron metabolism and lipid peroxidation are central drivers of ferroptosis, while the activation of antioxidant pathways, both dependent and independent on GPX4, serves as a defense mechanism against ferroptosis (Figure 2a). Elucidating the fundamental mechanisms governing ferroptosis initiation and regulation, especially the dynamic interplay between its drivers and defenses, establishes a crucial foundation for deciphering the mechanisms of action of ferroptosis inducers and inhibitors (Figure 2b).

### 2.1. Ferroptosis Drivers

#### 2.1.1. Lipid Peroxidation

Cellular uptake of polyunsaturated fatty acids (PUFAs) is mediated by fatty acid translocase (FAT) and fatty acid transport protein (FATP) [18]. Subsequently, ACSL4 catalyzes the esterification of long-chain PUFAs with CoA, followed by lysophosphatidylcholine acyltransferase 3 (LPCAT3), which incorporates them into lysophospholipids to form PUFA-phospholipids (PUFA-PLs). Then, Fe^2+^ or Fe^2+^-dependent enzymes like lipoxygenases (ALOXs) and cytochrome P450 oxidoreductase (POR) convert PUFA-PLs to toxic phospholipid hydroperoxides (PUFA-PL-OOHs), triggering membrane damage through free radical chain reactions [19]. Furthermore, lipophagic degradation of lipid droplets (LDs) releases free fatty acids, fueling lipid peroxidation and accelerating ferroptosis. Conversely, the enhancement of lipid storage mediated by tumor protein D52 (TPD52) can counteract RSL3-induced peroxidative stress and ferroptosis [20], indicating the importance of LD homeostasis in modulating ferroptosis susceptibility (Figure 2b).

#### 2.1.2. Disordered Iron Metabolism

Dietary iron, mainly as ferric iron (Fe^3+^), binds to transferrin (TF) and is transported into endosomes via transferrin receptor 1 (TfR1). Within endosomes, the metalloreductase STEAP3 reduces Fe^3+^ to Fe^2+^, which is then transported into the cytosol via solute carrier family 11 member 2 (SLC11A2/DMT1) to enter the labile iron pool (LIP) or be stored in ferritin [21]. Ferritin, composed of ferritin heavy chain 1 (FTH1) and ferritin light chain (FTL) subunits, plays a protective role by chelating and oxidizing Fe^2+^ to the more stable Fe^3+^ for storage. Nevertheless, this protection can be counteracted by nuclear receptor coactivator 4 (NCOA4)-mediated ferritinophagy, resulting in increased Fe^2+^ levels and promoting ferroptosis [22]. Cellular iron (Fe^2+^) export is primarily mediated by ferroportin (FPN) [23]. Furthermore, cellular iron homeostasis is dynamically regulated by iron regulatory proteins (IRP1/2), which interact with iron-responsive elements (IREs) in target mRNAs [24]. The functional outcome of this regulation depends on the position of the IRE within the mRNA. In iron-deficient conditions, IRPs bind to the 3′ untranslated region (UTR) of TfR1 mRNA to enhance its stability and promote iron import, while simultaneously binding to the 5′ UTR of ferritin mRNA to inhibit its translation and reduce iron storage. Conversely, in iron-replete conditions, IRPs dissociate from IREs, leading to TfR1 mRNA degradation and increased ferritin translation, thereby promoting iron storage [25,26,27]. Dysregulation at any point within the iron metabolism pathway can result in an accumulation of Fe^2+^ in the LIP. This excess Fe^2+^ reacts with hydrogen peroxide (H_2_O_2_) through the Fenton reaction, producing highly reactive oxygen species, including the hydroxyl radical (•OH) [28], which initiates lipid peroxidation through a non-enzymatic pathway, ultimately inducing ferroptosis (Figure 2).

The mitochondrial electron transport chain (particularly complexes I and III) serves as a major cellular source of ROS. Electron leakage generates superoxide anion (O_2_•^−^), which is then converted to H_2_O_2_ by superoxide dismutase (SOD) [29]. When H_2_O_2_ encounters intracellular free Fe^2+^, ROS are generated catalytically through the Fenton reaction. These radicals subsequently drive peroxidation of PUFA-PLs, making a critical event in the process of ferroptosis [29,30].

### 2.2. Defense Systems of Ferroptosis

#### 2.2.1. System x_c_^−^/GSH/GPX4 Antioxidant Pathway

The cystine–glutamate antiporter system (system x_c_^−^), consisting of the subunits SLC7A11 and SLC3A2, is integrated into the plasma membrane and plays a crucial role in cellular redox defense [31]. System x_c_^−^ mediates the 1:1 exchange of extracellular cystine for intracellular glutamate. The internalized cystine is reduced to cysteine, which is a vital precursor for the synthesis of reduced GSH [32,33]. GSH serves not only as an essential cofactor for GPX4 but also as a key factor for its antioxidant activity [34]. Distinct from other glutathione peroxidases (GPXs), GPX4 specifically reduces toxic L-OOHs to nontoxic L-OHs, while concurrently oxidizing GSH to glutathione disulfide (GSSG) [35].

#### 2.2.2. GPX4-Independent Antioxidant Pathways

Recent studies have identified three GPX4-independent pathways that exert antioxidant effects by scavenging free radicals, thereby inhibiting ferroptosis. These pathways are the NAD(P)H/FSP1/CoQ10 pathway, the DHODH/CoQ10 pathway, and the GCH1/BH4/DHFR pathway (Figure 2b) [36]. The ferroptosis suppressor protein 1 (FSP1), which utilizes nicotinamide adenine dinucleotide phosphate (NADPH) as an electron donor, reduces coenzyme Q10 (CoQ10) to ubiquinol (CoQ10H_2_). CoQ10H_2_ acts as a lipophilic radical-trapping antioxidant (RTA) that directly scavenges lipid radicals [37]. Dihydroorotate dehydrogenase (DHODH), located in the mitochondrial inner membrane, employs a similar radical-scavenging mechanism as FSP1 [38]. Tetrahydrobiopterin (BH_4_) is a potent RTA, and its radical-trapping activity relies on the regeneration mediated by dihydrofolate reductase (DHFR). In the biosynthesis of BH_4_, guanosine triphosphate cyclohydrolase 1 (GCH1) functions as the rate-limiting enzyme [39].

## 3. Ferroptosis Inducers

**Figure 3:**
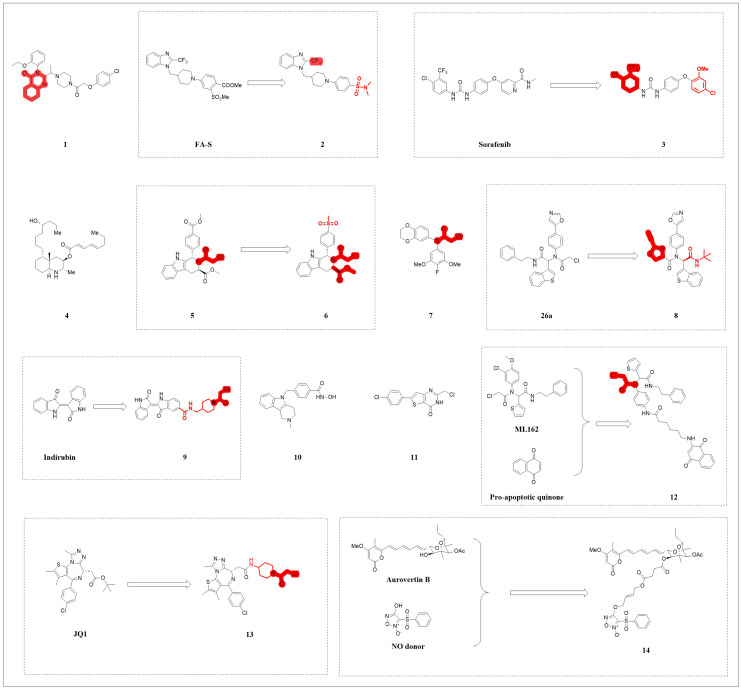
The structures of compounds **1**–**14**. Dashed boxes and arrows illustrate the structural optimization from precursors to derivatives. Red atoms represent the modified moieties in the derivatives. The red highlights indicate the identified pharmacophore of the compound, crucial for inducing ferroptosis.

**Figure 4:**
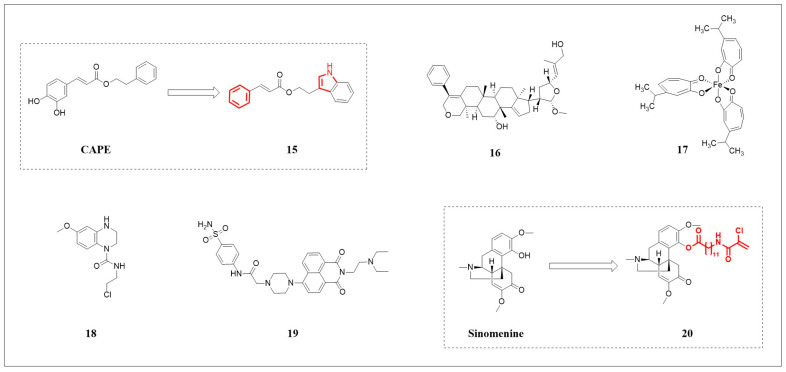
The structures of compounds **15**–**20**. Dashed boxes and arrows illustrate the structural optimization from precursors to derivatives. Red atoms represent the modified moieties in the derivatives.

**Figure 5:**
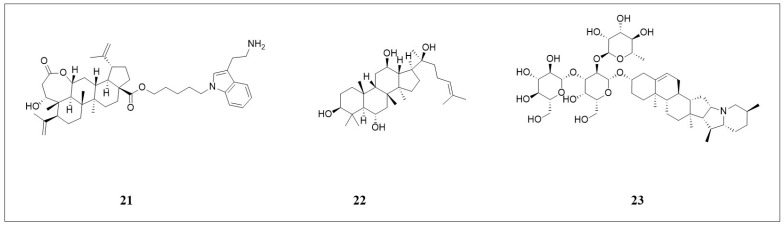
The structures of compounds **21**–**23**.

The chemical structures of ferroptosis inducers are depicted in Figure 3, Figure 4 and Figure 5. Based on their targets, ferroptosis inducers are classified into four categories: those targeting the system x_c_^−^/GSH/GPX4 axis, those targeting Fe^2+^ and ROS, those targeting lipid metabolism, and those targeting antioxidant systems independent of the GPX4.

### 3.1. Targeting the System x_c_^−^/GSH/GPX4 Axis

#### 3.1.1. Erastin and Its Combination Therapy

Erastin (Figure 3, compound **1**), the first identified ferroptosis inducer, triggers ferroptosis by inhibiting system x_c_^−^ and affecting the synthesis of GSH [1]. It also interacts with mitochondrial voltage-dependent anion channels 2/3 (VDAC2/3), altering channel properties and leading to mitochondrial dysfunction and increased ROS production, ultimately inducing ferroptosis [40]. The quinazolinone moiety within its molecular structure was identified as the active center. Nonetheless, its inadequate water solubility and metabolic stability issues constrain its in vivo utility [41]. Studies have revealed that specific tumor cells, notably intestinal cancer cells, display resistance to erastin-triggered ferroptosis [4,42]. Enhancing the sensitivity of colon cancer cells to erastin by using combination therapy may be beneficial for colon cancer treatment. Luteolin [43], mollugin [44], or sodium butyrate [45], used in combination with erastin, significantly inhibit the growth of colon cancer cells, demonstrating superior efficacy compared to erastin alone. Based on erastin inhibiting system x_c_^−^, mollugin and luteolin promote ferroptosis by inhibiting GPX4 expression. Sodium butyrate synergistically inhibits system x_c_^−^ activity with erastin by reducing CD44 expression, thereby decreasing the stability of the SLC7A11 subunit in the cell membrane. These findings underscore the importance of exploring the synergistic effects of drugs for ferroptosis-based cancer therapy.

#### 3.1.2. FA-S Derivative

Fang and colleagues [46] identified FA-S (Figure 3), a 2-(trifluoromethyl) benzimidazole derivative, as a novel ferroptosis inducer from a screening campaign against the human fibrosarcoma cell line HT-1080. Its scaffold differs from those of classical ferroptosis inducers, such as the quinazolinones represented by erastin or the chloroacetamides by RSL3. Structural modification of FA-S, introducing an amide group to replace the methyl ester moiety, improved its initial ferroptosis-inducing activity. Further studies revealed that the benzimidazole moiety with a trifluoromethyl group was crucial for maintaining the activity. Among the derivatives synthesized based on this, FA16 (Figure 3, compound **2**) significantly induced HT1080 cell ferroptosis by inhibiting system x_c_^−^ (IC_50_ = 1.26 μmol·L^−1^), and its activity was more than five-fold higher than that of FA-S (IC_50_ = 6.64 μmol·L^−1^). Compound **2** also significantly inhibited hepatocellular carcinoma growth in the BALB/c nude mouse model. Furthermore, compound **2** displayed superior metabolic stability in human and rat liver microsomes compared to erastin, underscoring its enhanced suitability for in vivo application and positioning it as a promising lead compound for further development [46].

#### 3.1.3. Sorafenib Derivative

Sorafenib (Figure 3) is an oral multi-kinase inhibitor primarily used to treat hepatocellular carcinoma and renal cell carcinoma by inhibiting the MEK/ERK signaling pathway [47]. The anticancer effects of sorafenib, not only from its kinase inhibition but also from off-target effects, promote ferroptosis via ROS generation or endoplasmic reticulum stress [48]. Notably, sorafenib induces ferroptosis by inhibiting system x_c_^−^. SAR analysis has revealed that the -CF_3_ and -Cl substitutions on the phenyl ring along the proximal ureido group are critical for the inhibition of system x_c_^−^ [41]. Based on SAR analysis, Kim and colleagues [49] synthesized sorafenib derivatives. Among these, compound **3** (Figure 3) significantly inhibited erastin-sensitive lung cancer cell line growth (including Calu1 and TD), and its anticancer efficacy was independent of the kinase inhibition pathway. Pharmacokinetic studies demonstrated that compound **3** had an oral bioavailability of 24%, comparable to that of sorafenib (38%~49%), indicating its feasibility as an oral formulation. In Calu1 lung cancer cell xenograft mouse models, compound **3** exhibited significant antitumor efficacy, validating its potential as an oral ferroptosis inducer. Precise structural optimization of the sorafenib derivative using site-selective affinity labeling technology redirected its anticancer mechanism from the primary pharmacophore of kinase inhibition to the off-target effect of ferroptosis induction [50]. Compound **3** represents a rational redesign of sorafenib that effectively decouples ferroptosis induction from its kinase-inhibitory effects. However, its translational path still requires careful consideration. A complete toxicological profile is yet to be established. Despite having similar oral bioavailability to sorafenib, enhancing solubility, metabolic stability, and the therapeutic window through lipid-based nanoparticles or solid dispersions warrants further exploration.

#### 3.1.4. Lepadin E/H

Wang and colleagues [51] synthesized a series of compounds featuring a decahydroquinoline (DHQ) motif and an ester side chain. Among them, lepadins E and H (Figure 3, compound **4**) exhibited significant cytotoxicity against the human cervical cancer HeLa cell line. Their bioactivity is attributed to the highly substituted DHQ group combined with a unique unsaturated acyl chain. Mechanistically, lepadin E/H orchestrated a multifaceted antitumor response by upregulating key proteins p53 and ACSL4 while downregulating the central ferroptosis defenders SLC7A11 and GPX4, thereby initiating ferroptosis. Notably, the elevated p53 also activated p21, resulting in cell cycle arrest at the G2/M phase. Through 2′,7′-dichlorofluorescein diacetate (DCFH-DA) staining, it was found that the treatment with lepadins E/H increased the ROS level, which not only drives ferroptosis but also exacerbates mitochondrial dysfunction and activates poly ADP-ribose polymerase (PARP), ultimately inducing apoptosis. This potent efficacy was corroborated in a B16F10 melanoma mouse model, where lepadin H treatment markedly suppressed tumor growth without observable toxicity to major organs, highlighting its promising in vivo safety profile. Lepadins E/H’s ability to engage multiple cell death pathways warrants further investigation to elucidate the synergistic mechanism fully and to assess their broader therapeutic potential against other cancer types.

In addition to the indirect strategy of inhibiting system x_c_^−^ to compromise GPX4 function, a more direct approach to targeting the system x_c_^−^/GSH/GPX4 axis is through the inhibition of GPX4. Direct GPX4 inhibition impairs its capacity to detoxify toxic PUFA-PL-OOHs, thereby triggering ferroptosis. The compounds discussed in the next part of this section are all direct GPX4 inhibitors.

#### 3.1.5. RSL3 Derivatives and RSL3 Combination Therapy

Yang and colleagues [52] discovered RSL3 (Figure 3, compound **5**) as a potent inducer of ferroptosis in a synthetic lethality screen targeting oncogenic Ras. The ferroptosis-inducing mechanism of RSL3 involves the alkylation of the selenocysteine residue (the active site of GPX4) by the chloroacetamide moiety, resulting in the irreversible inactivation of GPX4. However, its therapeutic application is hindered by metabolic instability [52,53]. Randolph and colleagues [54] found that the methyl ester group in the tetrahydro-β-carboline core of RSL3 is essential for its activity, while the methyl benzoate group has minimal impact. Structural modifications led to the development of RSL24 (Figure 3, compound **6**), with enhanced metabolic stability and water solubility. Nevertheless, in the WSU-DLCL2 diffuse large B-cell lymphoma mouse model, RSL24 demonstrated higher renal selectivity and limited efficacy against tumor growth, restricting its potential as a standalone GPX4 inhibitor. This challenge has steered the exploration of combination therapy strategies. Similar to erastin, RSL3 shows synergistic effects with various agents. For example, co-administration of the fatty acid amide hydrolase (FAAH) inhibitor URB597 with RSL3 enhances the ferroptosis-inducing activity of RSL3, leading to a substantial reduction in tumor volume in a nude mouse 786-O xenograft model [55].

Chen and colleagues [56] developed a novel GPX4 covalent inhibitor, C18 (Figure 3, compound **7**), by retaining the essential chloroacetamide warhead. In addition to covalently binding GPX4, compound **7** also forms an extensive hydrogen-bond network with key amino acid residues, including Trp136, Gly79, and Lys48. These interactions confer superior selectivity and improved pharmacokinetic properties compared to RSL3. It demonstrated potent inhibitory effects in triple-negative breast cancer (TNBC). In animal models, C18 (20 mg/kg) achieved a tumor growth inhibition (TGI) rate of 81.0% without eliciting toxicity in other organs, offering a novel therapeutic strategy for TNBC.

Previous studies optimized RSL3 to yield the precursor **26a** (Figure 3) (IC_50_ = 0.12 μmol·L^−1^, selectivity index, SI = 63); however, its pharmacokinetic properties and activity remained suboptimal [57]. Gu and colleagues [58] substituted the chloroacetyl moiety of **26a** with a 2-ethynylthiazole-4-carbonyl group through electrophilic warhead screening, obtaining highly active compounds. Refinement of the phenethyl and anilino structures led to the development of the chiral compound (R)-9i (Figure 3, compound **8**). It exhibited ultra-potent GPX4 inhibitory activity (IC_50_ = 0.3 nmol·L^−1^) and a remarkable selectivity index (SI = 24,933)—nearly 400-fold greater than that of the lead compound **26a**, underscoring the success of the structural optimization. Mechanistic studies confirmed that the potent activity of (R)-9i stems from its direct inhibition of GPX4, resulting in efficient induction of lipid peroxidation. In HT1080 cells, treatment with (R)-9i resulted in significantly higher levels of ROS and LPO compared to **26a** and RSL-3. Cotreatment with the ferroptosis inhibitor Fer-1 completely reversed these effects, confirming its ferroptosis-dependent cytotoxicity. Notably, despite its limited oral bioavailability, (R)-9i significantly suppressed tumor growth in an HT1080 xenograft model without inducing observable toxicity. In summary, these findings not only validate rational optimization of the covalent warhead as an effective strategy for enhancing the potency and selectivity of GPX4 inhibitors but also highlight that improving (R)-9i’s oral bioavailability through prodrug design represents a critical direction for future translation.

#### 3.1.6. Indirubin Derivative

Indirubin (Figure 3), the main active component of the traditional Chinese medicine Indigo Naturalis, is a bisindole alkaloid structurally similar to the ferroptosis inducer FIN56 (a non-covalent inhibitor of GPX4 that induces ferroptosis by promoting GPX4 degradation). Indirubin derivative (Figure 3, compound **9**) was synthesized by introducing an electrophilic group at the 5′-position of indirubin. As a novel GPX4 modulator, compound **9** differs from FIN56. It inhibits GPX4 activity through a dual mechanism: covalent binding to GPX4 via its chloroacetyl group and promotion of GPX4 ubiquitination. Compound **9** effectively induced ferroptosis in the human colon cancer cell line HCT-116, exhibiting the highest cytotoxicity among all derivatives (IC_50_ = 0.49 μmol·L^−1^), representing an approximately 40-fold enhancement compared to indirubin [59]. This study demonstrates that targeting GPX4 degradation, in combination with direct inhibition of its activity, represents a highly promising strategy for developing novel, potent ferroptosis inducers.

#### 3.1.7. Tubastatin A

Liu and colleagues [60] screened a small-molecule library and identified that Tubastatin A (Figure 3, compound **10**) significantly induced cell death in ferroptosis-sensitive cell lines but had no effect on resistant lines. In vitro studies demonstrated that compound **10** induces ferroptosis by directly inhibiting GPX4 activity, akin to RSL3. In TNBC MDA-MB-231 cells, radiotherapy upregulates GPX4 expression and suppresses its lysosomal degradation, bolstering cancer cell defenses against ferroptosis and radiotherapy. The combination of compound **10** with radiotherapy enhanced ferroptosis specificity and radiosensitivity by inhibiting GPX4. Despite potential hydroxamate-related side effects in Tubastatin A, its design offers a novel direction for developing GPX4 inhibitors. Future optimization strategies should prioritize modifying the hydroxamate group while preserving inhibitory efficacy to enhance its clinical utility.

#### 3.1.8. N6F11

Li and colleagues [61] identified N6F11 (Figure 3, compound **11**) as a potent ferroptosis inducer through a high-throughput screening in PANC1 pancreatic cancer cells. Mechanistically, N6F11-induced GPX4 degradation is linked to ubiquitination and is triggered by early-stage ubiquitination mediated by the intracellular E3 ligase tripartite motif containing 25 (TRIM25), rather than through autophagy. N6F11 induces GPX4 proteasomal degradation only upon binding to the PRY/SPRY (PS) domain of TRIM25. Given that TRIM25 is highly expressed in tumor cells but rarely in immune cells, N6F11 can specifically induce ferroptosis in cancer cells while protecting immune cells, thereby avoiding immune suppression side effects. This targeted mechanism positions N6F11 not only as a promising therapeutic candidate but also as a potential chemical probe for exploring TRIM25-dependent ferroptosis pathways in cancer biology.

#### 3.1.9. Dual-Targeting Compounds Inducing Ferroptosis and Apoptosis

Ma and colleagues [62] designed and synthesized a series of ML162-quinone conjugates based on pharmacophore hybridization and bioisosterism strategies, coupling the core skeleton of ML162 (Figure 3) with a pro-apoptotic quinone structure (Figure 3). This optimization yielded GIC-20 (Figure 3, compound **12**). Similar to ML162, GIC-20 can still covalently bind to the cysteine residue 46 of GPX4 through the chloroacetamide, directly inhibiting the activity of GPX4. Concurrently, GIC-20 induces proteasome-dependent degradation of GPX4 via a molecular glue mechanism. Although GIC-20 exhibited a slightly higher IC_50_ (1.6 μmol·L^−1^) than ML162 (0.6 μmol·L^−1^) in HT1080 cells, it activated a broader cell death response. Within the apoptosis pathway, GIC-20 activates apoptosis by upregulating the pro-apoptotic protein Bax and downregulating the anti-apoptotic protein Bcl-2. Annexin V/PI dual staining revealed that GIC-20-induced apoptosis was predominantly early-stage, whereas ML162 primarily induced late-stage apoptosis. This difference may stem from GIC-20 activating the caspase cascade via the mitochondrial pathway, while ML162 predominantly induces ferroptosis without involving apoptosis, indicating a fundamental difference in their apoptosis mechanisms. The therapeutic superiority of this dual-pathway activation was evidenced in an HT1080 xenograft model, where GIC-20 achieved a significant TGI rate of 63% relative to the blank control group without observable toxicity, effectively overcoming the limitations of single-mechanism agents.

Breast cancer has high mortality and incidence rates in women. TNBC, accounting for about 15% of cases, lacks estrogen receptor (ER), progesterone receptor (PR), and human epidermal growth factor receptor-2 (HER-2) expression. TNBC is more aggressive than other subtypes, with a high risk of metastasis, early recurrence, and poor prognosis [63]. The clinical management of TNBC faces challenges due to the scarcity of effective therapeutic targets and high rates of chemotherapy resistance. Bromodomain-containing protein 4 (BRD4), a key epigenetic regulator in TNBC, drives oncogene transcription and promotes tumor cell survival; however, the application of its inhibitor JQ1 (Figure 3) is often limited by drug resistance [64]. Given that TNBC cells exhibit high susceptibility to ferroptosis, which can synergize with apoptosis [65], concurrently targeting both pathways presents a promising therapeutic strategy. Building on this foundation, Ding and colleagues [66] modified JQ1 by introducing a chloroacetamide group at the acetate site via a rigid piperidine-based linker while retaining its BRD4-binding capability, thereby designing the dual-targeting compound **13** (Figure 3). This design yielded a dramatic enhancement in potency, with compound **13** exhibiting >38-fold improved antiproliferative activity (IC50 ≈ 0.5 μmol·L^−1^) over JQ1 in multiple TNBC cell lines. Mechanistically, compound **13** potently activated ferroptosis, as evidenced by significantly elevated lipid peroxidation and depleted GSH, while also robustly inducing apoptosis. This dual cell death induction translated into significant in vivo efficacy, suppressing tumor growth in an MDA-MB-231 xenograft model without apparent toxicity, underscoring its therapeutic potential.

In another approach to developing dual-targeting agents for TNBC, Ma and colleagues [67] sought to overcome the high toxicity and lipophilicity of the natural product aurovertin B (AVB, Figure 3) by designing hybrid molecules that conjugate AVB with NO donors (Figure 3). Through SAR analysis, compound **14** (Figure 3) emerged as the optimal candidate, demonstrating significantly enhanced antiproliferative potency against TNBC cell lines and a markedly improved selectivity index (SI > 79) compared to AVB, indicating a successful mitigation of general cytotoxicity. The potent activity of compound **14** was attributed to a dual mechanism of action, simultaneously inducing mitochondrial-mediated intrinsic apoptosis and triggering ferroptosis via the inhibition of key regulators GPX4 and FTH1. This dual cell death induction translated into profound in vivo efficacy, achieving near-complete tumor growth suppression in an MDA-MB-231 xenograft model. Notably, compound **14** also potently inhibited tumor metastasis in a 4T1 lung metastasis model, highlighting its multifaceted anti-TNBC capability.

In summary, the compounds discussed in this section (compounds **12**–**14**) collectively support a pivotal strategy in anticancer drug development: the design of dual-pathway inducers that concurrently initiate ferroptosis and apoptosis. This approach effectively addresses the limitations of single-mechanism therapies, such as drug resistance. Whether by directly inhibiting GPX4 or targeting its degradation, these compounds validate the synergistic potential of cross-regulation between ferroptosis and apoptosis. Future research should prioritize optimizing the therapeutic window of these drugs, particularly by tackling pharmacokinetic challenges such as oral bioavailability, and by identifying predictive biomarkers to select patient populations most likely to benefit from this effective combination therapy.

### 3.2. Targeting Fe^2+^ and ROS

Nuclear factor erythroid 2-related factor 2 (Nrf2) is a crucial regulator of the cellular antioxidant response, typically controlled by Kelch-like ECH-associated protein 1 (Keap1) in the cytoplasm. Keap1 negatively modulates Nrf2 by promoting its ubiquitination and subsequent proteasomal degradation. Under oxidative stress, Nrf2 dissociates from Keap1 and relocates to the nucleus, where it binds to antioxidant response elements (AREs). This binding triggers the transcription of genes related to ARE and detoxification, thereby enhancing cellular antioxidant defenses [68]. Nrf2-regulated target gene products include SLC7A11, GPX4, and heme oxygenase-1 (HO-1) (Figure 2b). HO-1 possesses dual functions, including cytoprotection and promotion of ferroptosis, with its effects dependent on the expression level and cellular context. Under conditions of moderate activation, HO-1 primarily exerts its cytoprotective effect by facilitating the clearance of ROS. In contrast, excessive or sustained upregulation of HO-1 significantly increases the labile Fe^2+^, leading to elevated ROS levels that overwhelm the antioxidant defense system and thereby promote the onset of ferroptosis [69].

#### 3.2.1. Caffeic Acid Phenethyl Ester (CAPE) Derivative

CAPE (Figure 4) is a natural inducer of HO-1 [70]. Under electrophilic conditions, it activates the thiol group, triggering a 1,4-Michael addition reaction, which promotes the dissociation of Keap1 from Nrf2, excessively activating the Nrf2/HO-1 pathway. This upregulation culminates in Fe^2+^ accumulation and elevated ROS, thereby driving ferroptosis [71]. To enhance the potency of CAPE, Consoli and colleagues [72] selectively modified the catechol moiety and the phenethyl ester aromatic ring through SAR analysis. This effort yielded a series of derivatives, among which compound **15** (Figure 4) exhibited the most potent cytotoxicity, over threefold greater than that of the parent compound CAPE against MDA-MB-231 cells. Preliminary computational assessments of compound **15** predicted favorable gastrointestinal absorption and moderate water solubility, suggesting a promising pharmacokinetic profile for its further development as a clinical candidate.

#### 3.2.2. Pacidusin B

Zhu and colleagues [73] extracted and identified four triterpenoids from the leaves of *Phyllanthus acidus*, among which pacidusin B (Figure 4, compound **16**) showed significant cytotoxicity in HT1080 cells. Mechanistic investigation revealed that its potency stems from the excessive activation of the PERK/Nrf2/HO-1 pathway, resulting in a dramatic over 40-fold upregulation of HO-1. This aberrant induction resulted in Fe^2+^ overload and oxidative stress, thereby triggering ferroptosis. This was corroborated by the concentration-dependent accumulation of LPO upon treatment with compound **16**. Additionally, molecular docking suggested a potential inhibitory interaction with system x_c_^−^, similar to erastin. Collectively, these findings position pacidusin B as a natural ferroptosis inducer that primarily acts through hyperactivation of the Nrf2/HO-1 axis, exemplifying the context-dependent pro-death function of this pathway.

#### 3.2.3. Hinokitiol-Iron Complex (Fe(hino)_3_)

In MDA-MB-231 cells, the combination treatment of hinokitiol (Hino) with iron (FeCl_3_) exhibited significantly greater cytotoxicity than that with either agent alone. The β-diketone structure of Hino allows it to form an Fe(hino)_3_ complex (Figure 4, compound **17**). This complex exhibited a characteristic absorption peak at 425 nm in UV-Vis spectroscopy, indicating its formation in cells at a 3:1 ratio. Compound **17**, with lipophilicity and redox characteristics, can penetrate the cell membrane. It primarily acts as a redox-active complex to exert pro-oxidant effects via the Fenton reaction and concurrently depletes GSH. Additionally, it plays a secondary role by releasing Fe^3+^, which, upon intracellular reduction to the ferrous state (Fe^2+^), could further exacerbate the Fenton reaction, contributing to ROS accumulation, lipid peroxidation, and ultimately inducing ferroptosis in TNBC cells [74].

#### 3.2.4. Urea Derivative

Based on the principle of bioisosterism, Liang and colleagues [75] designed and synthesized novel urea derivatives utilizing tetrahydroquinoxaline as the skeleton and replacing the linking group with a urea group. In antitumor proliferation activity tests against five human cancer cell lines, the urea derivative compound **18** (Figure 4) demonstrated potent and selective anti-proliferative activity against colon cancer HT-29 cells (IC_50_ = 1.97 μmol·L^−1^). The anticancer activity of compound **18** primarily stemmed from its profound disruption of cellular redox homeostasis, triggering a dramatic surge of ROS. This oxidative burst has been demonstrated to be reversed by the scavenger N-acetyl-L-cysteine (NAC). As a key upstream event, it initiates both ferroptosis and autophagy—two distinct cell death processes that operate independently of each other. Thus, compound **18** acts as a potent inducer capable of triggering parallel cell death responses centered on ROS generation. Nevertheless, the precise molecular targets and signaling events upstream of ROS accumulation warrant further investigation.

#### 3.2.5. Benzenesulfonamide Derivatives

Prior work from Zhang’s research group suggested that 4-(N-substituted piperazine)-1,8-naphthalamides exhibit antitumor potential [76]. Building on this finding, Liang and colleagues [77] from the same group later designed and synthesized a series of 1,8-naphthalimide piperazinamide-based benzenesulfonamides, carbonic anhydrase IX (CA IX) inhibitors. CA IX, induced by HIF-1α, is overexpressed in hypoxic regions of solid tumors and promotes malignant phenotypes. Among these derivatives, compound **19** (Figure 4) demonstrated significant inhibitory activity and selectivity against CA IX in MDA-MB-231 cells, particularly under hypoxic conditions (IC_50_ = 5.67 μmol·L^−1^), outperforming normoxic conditions (IC_50_ = 19.76 μmol·L^−1^). Compound **19** exerts a multi-mechanistic antitumor effect by inhibiting cell migration, inducing cell cycle arrest and apoptosis. Notably, it also triggered ferroptosis, primarily associated with mitochondrial dysfunction—as indicated by the loss of mitochondrial membrane potential (MMP)—and a consequential surge in overall cellular ROS. While mitochondrial impairment appears to be a significant contributor to oxidative stress, the potential involvement of other sources contributing to the elevated ROS warrants further investigation. Therefore, compound **19** represents a promising candidate that uniquely integrates CA IX inhibition with the induction of ferroptosis, offering a coordinated strategy to target hypoxic tumor niches. Future work should focus on delineating the detailed mechanism underlying its ferroptosis-inducing activity.

#### 3.2.6. Sinomenine Derivative

Zhu and colleagues [78] synthesized novel derivatives based on the antitumor activity of sinomenine (Figure 4) by introducing electrophilic groups to promote ROS generation and long-chain fatty acids to enhance cytotoxicity. In HCT-116 and HT-29 cells, sinomenine derivative compound **20** (Figure 4) exhibited significantly superior cytotoxicity (IC_50_ = 5.87 μmol·L^−1^) compared to sinomenine (IC_50_ = 1.76 mmol·L^−1^). As mentioned above in Section 2, “Molecular Mechanisms of Ferroptosis”, ferritin is transported to lysosomes mediated by NCOA4. The degradation of ferritin by lysosomes triggers ferritinophagy, leading to an increase in the concentration of Fe^2+^ and inducing ferroptosis (Figure 2b). Compound **20** acts on this pathway to induce ferroptosis. However, **20** does not affect the expression of NCOA4. Instead, it enhances the interaction between NCOA4 and FTH1, promotes the degradation of FTH1 in lysosomes, releases Fe^2+^, accumulates ROS, and ultimately induces ferroptosis.

### 3.3. Targeting Lipid Metabolism

#### 3.3.1. Seco-Lupane Triterpene Derivative

Wang and colleagues [79] synthesized novel seco-lupane triterpene derivatives and identified compound **21** (Figure 5) as exhibiting significant inhibitory effects on hepatocellular carcinoma HepG2 cells (IC_50_ = 0.97 μmol·L^−1^). Compound **21** induces ferroptosis by upregulating ACSL4, downregulating GPX4 expression, and increasing lipid peroxidation. Additionally, **21** binds to the structural domain of cyclin D1 protein and suppresses its expression, thereby inhibiting the CDK4/CDK6-mediated phosphorylation of retinoblastoma protein (Rb) and ultimately inducing G1 phase cell cycle arrest in HepG2 cells. Compound **21** demonstrated superior cytotoxicity against HepG2 cells at low concentrations compared to several first-line clinical drugs, including doxorubicin, docetaxel, etoposide, gemcitabine, and 5-fluorouracil. These findings indicate that **21** has potential as a novel therapeutic agent for liver cancer.

#### 3.3.2. (20S)-Protopanaxatriol

(20S)-Protopanaxatriol [(20S)-PPT, Figure 5, compound **22**], a triterpenoid isolated from *Panax notoginseng*, specifically enhances the sensitivity of osteosarcoma cells to ferroptosis inducers by upregulating ACSL4 transcription [80]. Its core mechanism relies on ACSL4-mediated generation of lipid peroxidation substrates. Western blot analysis revealed that (20S)-PPT selectively elevates ACSL4 protein levels without significantly affecting GPX4, SLC7A11, or FSP1. Knockdown of ACSL4 using specific shRNA completely reversed the sensitizing effect of (20S)-PPT. In vitro, co-treatment with (20S)-PPT and ferroptosis inducers (RSL3, ML162, imidazole ketone erastin [IKE]) resulted in significantly reduced viability of osteosarcoma U2OS and MG63 cells compared to the inducer alone, as assessed with MTT assay, while (20 S)-PPT alone showed no toxicity. This synergy was robustly validated in a U2OS xenograft model, where the combination of (20S)-PPT and IKE achieved a superior 74% reduction in tumor volume compared to IKE monotherapy (42%), accompanied by confirmed upregulation of ACSL4 in tumor tissues. This study highlights a promising low-toxicity strategy that leverages a natural product to potentiate ferroptosis. However, the strict dependence on ACSL4 expression underscores the need for patient stratification biomarkers. Future efforts should focus on elucidating the upstream transcriptional mechanism and improving the compound’s bioavailability for clinical translation.

#### 3.3.3. Solanine

Solanine (Figure 5, compound **23**) is a phytochemical isolated from traditional Chinese medicine, and its anticancer effects have been reported. In the colon cancer cell lines HCT116 and SW480 treated with solanine, the expression of arachidonate 12-lipoxygenase B (ALOX12B) and arachidonate 5-lipoxygenase (ALOX5) was significantly upregulated in a dose-dependent manner. Among them, ALOX12B was more significantly upregulated, promoting lipid peroxidation. Silencing adenylate cyclase 4 (ADCY4) could reduce the stability of ALOX12B and inhibit its overexpression, which indicates that ADCY4 is crucial for maintaining the expression and stability of ALOX12B, essential for the effective execution of ferroptosis in colon cancer cells treated with solanine [81].

### 3.4. Targeting GPX4-Independent Antioxidant Pathways

#### 3.4.1. Ferroptosis Sensitizer 1 (FSEN1)

**Figure 6:**
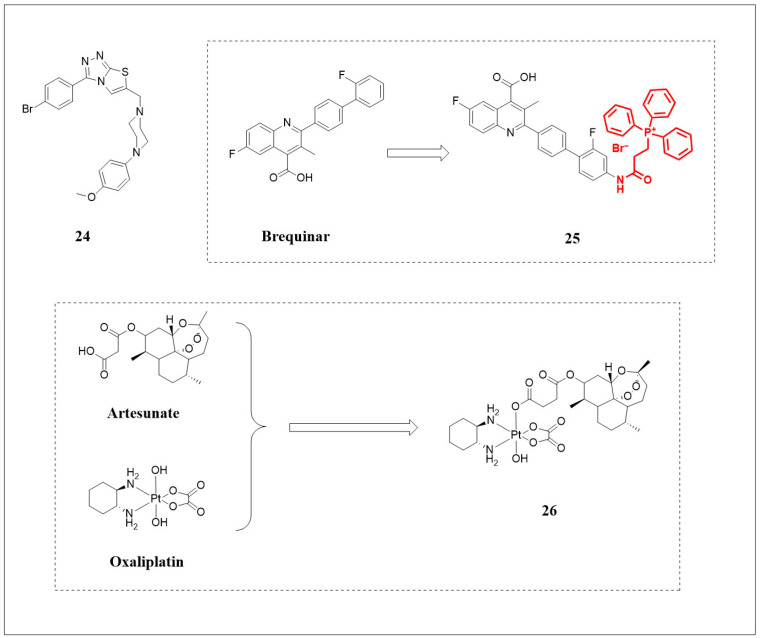
The structures of compounds **24**–**26**. Dashed boxes and arrows illustrate the structural optimization from precursors to derivatives. Red atoms represent the modified moieties in the derivatives.

Using an in vitro FSP1 activity assay, Hendricks and colleagues [82] screened 168 FSP1 inhibitors and identified FSEN1 (Figure 6, compound **24**) as exhibiting the strongest inhibitory effect due to its unique disubstituted [1,2,4] triazolo-thiazole core structure. Compound **24** acts as a non-competitive inhibitor, requiring FSP1 to be bound to its substrates (NADH/CoQ), thereby allowing **24** to bind and form an inactive complex. Nrf2 is involved in the transcriptional regulation of FSP1. The therapeutic relevance of targeting FSP1 is highlighted in certain contexts, such as Keap1-mutant lung cancers (e.g., H460, A549), where FSP1 is highly expressed and GPX4 levels are low, suggesting that FSP1 inhibition could be a particularly effective strategy in these tumors [83].

#### 3.4.2. Brequinar Derivative

Brequinar (BRQ, Figure 6) is a widely used DHODH inhibitor. To enhance the efficacy of BRQ in targeting DHODH, Hai and colleagues [84] synthesized the derivative compound **25** (Figure 6) by introducing the mitochondrial targeting group triphenylphosphine (TPP, Figure 6) into BRQ. Compound **25** induces ROS accumulation and promotes lipid peroxidation by inhibiting DHODH expression and impairing mitochondrial function. Compared to the parent compound BRQ, compound **25** exhibited 5.6-fold higher cytotoxicity against two melanoma cell lines, B16F10 and A375. Furthermore, compound **25** modulates the expression of mitochondrial-associated proteins, including methylenetetrahydrofolate dehydrogenase 2 (MTHFD2) and lactamase beta (LACTB) in metabolic pathways and significantly downregulates PD-L1, thereby alleviating immunosuppression. In a B16F10 mouse model, the compound **25** demonstrated superior antitumor activity compared to BRQ, without causing significant toxicity to major organs. In summary, compound **25** serves as a pioneering example of how rational mitochondrial targeting can significantly enhance the efficacy of metabolic inhibitors, such as brequinar. Its ability to concurrently disrupt mitochondrial function to induce ferroptosis and modulate immune checkpoints opens a promising therapeutic avenue, particularly for tumors that rely on mitochondrial metabolism.

#### 3.4.3. Oxaliplatin–Artesunate Complex (OART)

Artesunate (Figure 6) promotes ferroptosis by inducing ferritinophagy to release free Fe^2+^ and promoting ROS accumulation [85]. Fan and colleagues [86] developed a novel ferroptosis inducer, the oxaliplatin–artesunate complex (OART, Figure 6, compound **26**), by conjugating artesunate with the chemotherapeutic drug oxaliplatin (Figure 6). Compound **26** exhibited significant in vitro and in vivo bioactivity. Compound **26** showed superior cytotoxicity against mouse breast cancer 4T1 cells (IC_50_ = 1.26 μmol·L^−1^) compared to either parent drug alone, demonstrating the success of this combinatorial approach. Compound **26** induces ferroptosis by causing an intracellular redox imbalance through dual inhibition of the ferroptosis defense system. Specifically, it simultaneously disrupts the GPX4 pathway by depleting GSH and downregulating GPX4/SLC7A11, while also impairing the mitochondrial DHODH-CoQ10 axis, leading to massive lipid peroxidation specifically within mitochondria, as confirmed by the C11-BODIPY probe.

**Figure 7:**
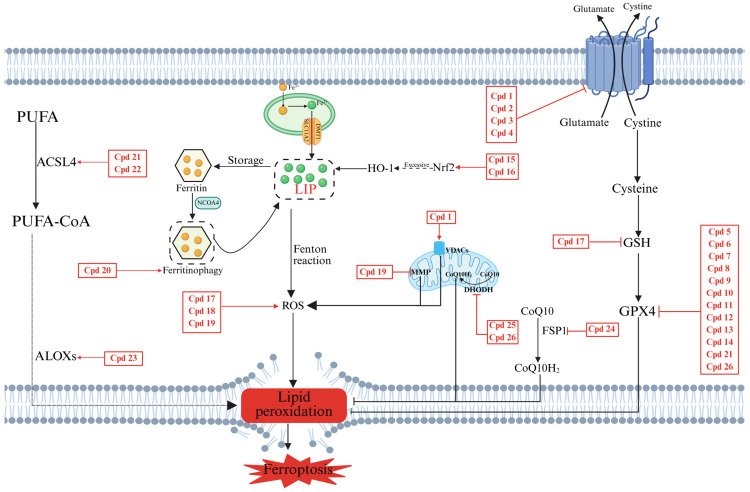
Schematic illustration of the modulatory mechanism of ferroptosis inducers. The diagram summarizes how the discussed small-molecule inducers trigger relevant ferroptosis pathways. Compound labels (e.g., Cpd 1) correspond to the numbers assigned in the text, Figure 3, Figure 4, Figure 5 and Figure 6, and Table 1. Created in Biorender. Chen, J. (2025) https://BioRender.com/gf27kb1 (accessed on 16 November 2025).

These studies not only provide a crucial molecular basis for the anticancer treatment of ferroptosis inducers but also promote the development of novel therapeutic strategies. To visually illustrate how these ferroptosis inducers target key pathways, we have integrated their mechanisms of action into a schematic diagram (Figure 7). Table 1 offers a comprehensive summary of their classification, core mechanisms of action, and related disease model studies.

## 4. Ferroptosis Inhibitors

Given that a target-based classification is less applicable to the current landscape of ferroptosis inhibitors, we have organized them according to their origin. This approach effectively distinguishes between the well-defined structures of synthetic inhibitors and the diverse pharmacophores of natural inhibitors, providing a clear framework for discussion.

### 4.1. Synthetic Inhibitors

#### 4.1.1. Arylamine Inhibitors

**Figure 8:**
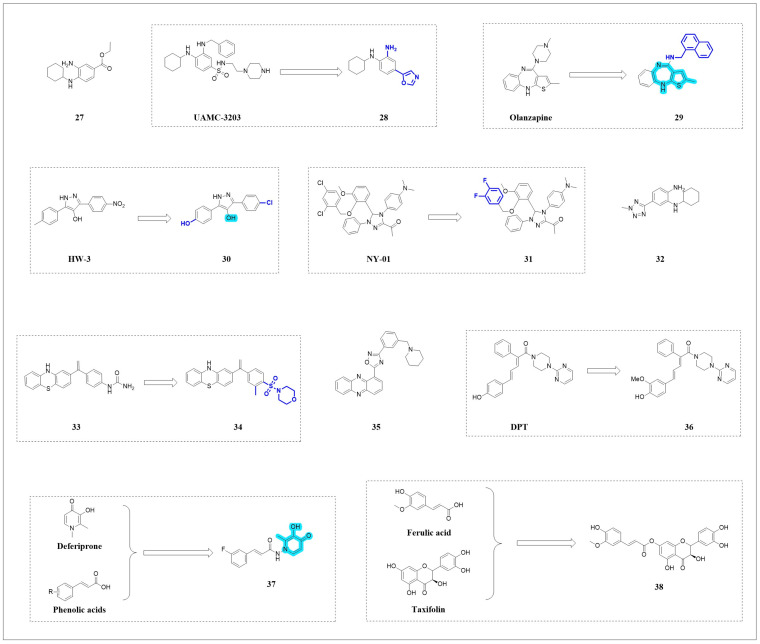
The structures of compounds **27**–**38**. Dashed boxes and arrows illustrate the structural optimization from precursors to derivatives. Blue atoms represent the modified moieties in the derivatives. The blue highlights indicate the identified pharmacophore of the compound, crucial for inhibiting ferroptosis.

Fer-1 (Figure 8, compound **27**) and liproxstatin-1 (Lip-1) are early identified ferroptosis inhibitors. Fer-1 functions by capturing free radicals and inhibiting lipid peroxidation. Fer-1 can inhibit ferroptosis in HT-1080 cells induced by erastin or RSL3. However, Fer-1 is unable to inhibit ferroptosis triggered by H_2_O_2_ or rotenone [1]. The oxindole–curcumin hybrid compound GIF-2165X-G1 [88] and bisbenzylisoquinoline compounds [89] inhibit ferroptosis induced by erastin or RSL3 but fail to reduce ROS production caused by rotenone, suggesting that the cytoprotective effects of antioxidants may depend on the specific source of oxidative stress.

In 2018, Devisscher and colleagues [90] developed a higher-activity analogue, UAMC-3203 (Figure 8), based on Fer-1. However, its application as a lead compound was limited by low oral bioavailability (F% = 7%) and insufficient central nervous system (CNS) penetration. To overcome these limitations, Scarpellini and colleagues [91] subsequently designed novel lipophilic RTAs through strategies including reducing molecular size, decreasing alkalinity, and minimizing hydrogen-bond donor groups. This led to the development of the ferroptosis inhibitor UAMC-4821 (Figure 8, compound **28**). Compound **28** (IC_50_ = 5.2 nmol·L^−1^) exhibited comparable ferroptosis-inhibiting activity to UAMC-3203 (IC50 = 9.2 nmol·L^−1^) in the ML162-induced ferroptosis model of HT1080 cells. Crucially, UAMC-4821 exhibited a superior pharmacokinetic profile, characterized by significantly reduced plasma clearance and measurable brain exposure, resulting in a high oral bioavailability of 63%. This marked enhancement in its drug-like properties underscores its potential for testing in models of CNS disorders. This study successfully balanced radical-trapping potency with pharmacokinetic optimization, yielding a high-bioavailability candidate for treating ferroptosis-associated NDDs.

#### 4.1.2. N-Heterocyclic Inhibitors

Jiang and colleagues [92] screened an FDA-approved drug library and discovered olanzapine (Figure 8) as a potent ferroptosis inhibitor in RSL3-induced mouse hippocampal neuronal HT22 cells (EC_50_ = 1.2 μmol·L^−1^). Based on SAR analysis of olanzapine, they synthesized thienobenzodiazepine derivatives, among which compound **29** (Figure 8) demonstrated a 16-fold improvement in ferroptosis inhibition (EC50 = 0.074 μmol·L^−1^) and exhibited low cytotoxicity (CC_50_ = 18.8 μmol·L^−1^). Similar to Fer-1, compound **29** inhibits ferroptosis as RTA, with its thienobenzodiazepine scaffold being critical for its activity.

In the RSL3-induced HT-1080 cell ferroptosis model, the 4-hydroxyl pyrazole derivative HW-3 (Figure 8) was identified as a potent ferroptosis inhibitor through phenotypic screening. Subsequent optimization of this scaffold yielded compound **30** (Figure 8), which demonstrated a remarkable 14-fold increase in anti-ferroptosis potency (EC_50_ = 8.6 nmol·L^−1^) compared to HW-3 (EC_50_ = 120.1 nmol·L^−1^). Mechanistically, compound **30** does not act via iron chelation but functions as a potent RTA. This was evidenced by its direct scavenging of 2,2-diphenyl-1-picrylhydrazyl (DPPH) radicals and its superior antioxidant activity relative to Fer-1, confirming that it inhibits the ferroptosis cascade by directly neutralizing lipid radicals to suppress lipid peroxidation. The 4-hydroxy group is essential for its activity [93].

Zhang and colleagues [94] constructed a library of 900 molecules based on the concept of pseudo-natural product design. Using an RSL3-induced 786-O cell ferroptosis model, they performed phenotypic screening and first identified the 1,2,4-triazole compound NY-01 (Figure 8) as a potent ferroptosis inhibitor (EC_50_ = 0.26 μmol·L^−1^). Through SAR analysis and optimization, NY-26 (Figure 8, compound **31**) was developed, with its activity increased by 4-fold (EC_50_ = 62 nmol·L^−1^). Due to its inherent free radical-trapping antioxidant capacity, NY-26 can inhibit ferroptosis. C11-BODIPY fluorescent probe analysis demonstrated its rapid clearance of RSL3-induced ROS accumulation, with efficiency comparable to the classical antioxidant Fer-1. In a concanavalin A-induced acute liver injury model, pretreatment with NY-26 significantly reduced serum alanine aminotransferase (ALT) and aspartate aminotransferase (AST) levels, thereby alleviating histopathological damage in liver tissue.

Starting from the LPO scavenging activity of phenyltetrazolium compounds, Lu and colleagues [95] performed structural optimization focused on radical-trapping capacity and phospholipid bilayer retention. It was found that the 2-methyl-2H-tetrazolyl group could mimic the phosphate group of the hydrophilic head of phospholipids, and a novel ferroptosis inhibitor compound **32** (Figure 8) was developed. Compound **32** exhibited potent anti-lipid peroxidation activity (IC_50_ = 22 nmol·L^−1^) in the erastin-induced HT1080 cells model. Its core mechanism of free radical scavenging was validated via the DPPH assay, where it significantly outperformed the classic inhibitor Fer-1. Pharmacokinetic evaluation revealed favorable plasma exposure but limited brain distribution upon intravenous administration, indicating a primary distribution to peripheral tissues like the liver and kidneys. Despite this, compound **32** demonstrated significant efficacy in a transient middle cerebral artery occlusion (tMCAO) model, effectively reducing cerebral infarct volume, alleviating neurological deficits, and counteracting the downregulation of key ferroptosis-related proteins (GPX4/SLC7A11).

#### 4.1.3. Phenothiazine and Phenazine Inhibitors

You and colleagues [96] synthesized a phenothiazine-based ferroptosis inhibitor, compound **33** (Figure 8), which demonstrated high efficacy in the cellular models and low affinity for the *human Ether-a-go-go-related gene* (hERG) K^+^ channel, thereby mitigating potential cardiotoxicity risks. Its safety and therapeutic potential were further validated in a model of doxorubicin-induced cardiomyopathy. To advance the scaffold, Bai and colleagues [97] introduced sulfonyl groups to diminish hERG inhibition (IC_50_ > 30 μmol·L^−1^), incorporated piperazine analogues to enhance anti-ferroptosis activity, and developed sulfonamide phenothiazine inhibitors. Among these, compound **34** (Figure 8) showed ultrahigh potency (EC_50_ = 1 nmol·L^−1^) in erastin-treated rat pheochromocytoma cell line PC12. The mechanism of both compounds is rooted in their intrinsic capacity to act as radical-trapping antioxidants, directly scavenging free radicals as confirmed by DPPH assays, where they outperformed Fer-1, thereby effectively suppressing lipid peroxidation. In a rat spinal cord injury (SCI) model, compound **34** significantly improved motor function recovery, reduced tissue cavitation, and reversed SCI-induced markers of ferroptosis.

The aforementioned RTAs, represented by Fer-1 and its derivatives, effectively terminate the chain reaction of lipid peroxidation by directly neutralizing lipid radicals, offering a direct and highly efficient strategy for inhibiting ferroptosis. However, this approach primarily targets the terminal effects of ferroptosis, making it challenging to regulate the upstream pathological signals that drive lipid peroxidation—such as GPX4 inactivation or disordered iron metabolism. This has prompted increased exploration of upstream regulatory strategies within the research field. Specifically, targeting the expression or activity of key proteins in the ferroptosis pathway enables intervention at the source of ferroptosis. This approach will be thoroughly discussed below. Future ideal therapeutic strategies may require combining the strengths of both approaches to achieve synergistic treatment.

Structurally analogous to phenothiazine, phenazine compounds feature an N/N bridge between benzene rings, distinct from the S/N-bridged phenothiazine scaffold. Wu and colleagues [98] synthesized a phenazine derivative compound **35** (Figure 8) as a novel non-classical ferroptosis inhibitor. It demonstrated significant inhibitory efficacy in an erastin-induced ferroptosis model using ES-2 human ovarian clear cell carcinoma cells (EC_50_ = 0.7 nmol·L^−1^, which is 1000-fold more potent than Fer-1). Compound **35** specifically suppresses ferroptosis by modulating the ferritinophagy pathway. In erastin-treated ES-2 cells, it upregulated NCOA4 and ferritin expression, blocking iron release via ferritin-lysosome degradation. In an acetaminophen-induced liver injury model, compound **35** effectively attenuated histopathological damage, reduced serum ALT/AST levels, and hepatic MDA, outperforming Fer-1. By targeting NCOA4-regulated iron homeostasis, this work provides a strategy for developing non-canonical ferroptosis inhibitors that circumvent the side effects of traditional antioxidant/iron-chelating therapies.

#### 4.1.4. Diphenylbutenyl Inhibitors

Fang and colleagues [99] identified a structurally novel ferroptosis inhibitor, the diphenylbutenyl derivative DPT (Figure 8), suppressing erastin-induced ferroptosis in HT22 cells (EC_50_ = 12.0 μmol·L^−1^). Based on the DPT scaffold, derivatives were developed, among which compound **36** (Figure 8) exhibited a nearly 7-fold improvement in ferroptosis-inhibiting activity (EC_50_ = 1.7 μmol·L^−1^). Compound **36** inhibits ferroptosis by elevating protein levels of FSP1 and activating the FSP1-CoQ10 pathway. Compound **36** can penetrate the blood–brain barrier (BBB) and reduce brain injury in a rat model of ischemic stroke, demonstrating neuroprotective effects, with potential for further development as a therapeutic agent for neurological disorders.

#### 4.1.5. Hybrid Inhibitors

Zhang and colleagues [100] integrated the antioxidant scaffold of phenolic acids (Figure 8) and the iron-chelating pharmacophore of deferiprone (DFP, Figure 8) to develop novel cinnamamide–hydroxypyridinone derivatives. In the erastin-induced HT22 model, compound **37** (Figure 8, EC_50_ = 14.89 μmol·L^−1^) demonstrated anti-ferroptosis effects nearly 10 times greater than those of DFP (EC_50_ = 164.6 μmol·L^−1^). Moreover, compound **37** significantly alleviated cisplatin-induced nephrotoxicity in a human embryonic kidney HEK293T cell acute kidney injury model, with its mechanisms of action at least partially involving reduction of intracellular Fe^2+^ and scavenging of free radicals. While the results are promising, the precise mechanism underlying its ferroptosis inhibition requires further elucidation, and its efficacy warrants additional enhancement in future studies.

Natural antioxidants, such as taxifolin, quercetin, cinnamic acid, and ferulic acid, exhibit a potent radical-scavenging capacity [101]. Guenther and colleagues [102] synthesized four phenolic acid–flavonoid hybrid compounds via ester/amide bonds. These hybrids effectively counteracted RSL3-, erastin-, and glutamate-induced ferroptosis in HT22 cells, with the ferulic acid–taxifolin conjugate UW-MD-190 (Figure 8, compound **38**) showing remarkable protection (EC_50_ < 0.5 μmol·L^−1^). Unlike conventional antioxidants, compound **38** inhibits ferroptosis by suppressing mitochondrial complex I activity and reducing mitochondrial respiration rather than through direct radical neutralization.

### 4.2. Natural Inhibitors

Recent studies have revealed the ferroptosis-inhibitory potential of numerous natural compounds, primarily derived from plants (especially traditional Chinese herbs) or endogenous molecules produced by cells themselves.

#### 4.2.1. Polyphenolic Inhibitors

**Figure 9:**
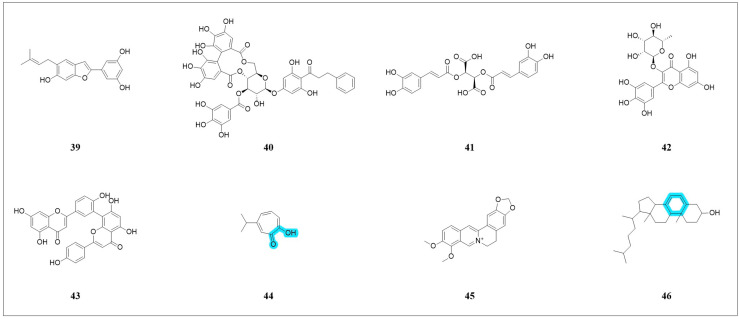
The structures of compounds **39**–**46**. The blue highlights indicate the identified pharmacophore of the compound, crucial for inhibiting ferroptosis.

Mulberry leaves, as a traditional Chinese medicine (TCM), have been found to possess diverse pharmacological activities, including neuroprotection. Wen and colleagues [103] isolated four prenylated phenolics from mulberry leaves, with moracin N (Figure 9, compound **39**) being the most abundant. It demonstrated significant protection against ferroptosis in HT22 cells (EC_50_ < 0.50 μmol·L^−1^) by inhibiting GSH depletion, preventing GPX4 inactivation, reducing ROS overproduction and Fe^2+^ accumulation while enhancing intracellular antioxidant enzyme activity.

Thonningianin A (ThA, Figure 9, compound **40**), a natural ellagic tannin polyphenol, inhibits ferroptosis via triple modulation of the Keap1/Nrf2 pathway: (a) direct Keap1 binding to disrupt Nrf2 interaction, (b) Atg7-dependent autophagic degradation of Keap1, and (c) p62-mediated Keap1 degradation—collectively upregulating antioxidant gene HO-1 [104]. Recent research has further expanded our understanding of its mechanism. As reported in [105], ThA binds to GPX4, enhances the AMPK/Nrf2 signaling pathway, and stimulates the activation of GPX4, thereby effectively inhibiting ferroptosis. In a cellular model of AD, ThA demonstrated significant neuroprotective effects, reducing cell mortality with potency comparable to the inhibitor liproxstatin-1 (Lip-1). This protective effect was corroborated in transgenic C. *elegans* AD models, where ThA alleviated disease-related phenotypes and reduced key markers of ferroptosis, including ROS, LPO, and Fe^2+^. Most importantly, after knocking down the expression of *aak-2* (equivalent to human AMPK) and *skn-1* (equivalent to mammalian Nrf2) in *C. elegans* using RNAi, the anti-ferroptosis effect of ThA was significantly attenuated, directly demonstrating that it inhibited ferroptosis via the AMPK/Nrf2/GPX4 pathway.

Chicoric acid (CA, Figure 9, Compound **41**), an immunologically active constituent extracted from chicory and *Echinacea purpurea*, alleviates asthma progression by inhibiting the key ferroptosis regulator ALOX15 [106]. It functions by binding to ALOX15 and blocking its ability to drive lipid peroxidation. In cellular and murine models of asthma, CA treatment effectively suppressed a broad spectrum of ferroptosis markers, including ROS and Fe^2+^, and restored the expression of protective proteins. ALOX15-specific knockdown mimicked the inhibitory effect of CA on lipid peroxidation, further validating its target specificity. In vivo, CA not only mitigated hallmark pathological features of asthma, such as inflammation and fibrosis, but also modulated the expression of core ferroptosis regulators GPX4 and SLC7A11, positioning it as a multi-faceted inhibitor with therapeutic potential for asthma and other ALOX15-associated diseases [106].

Curcumin activates the Nrf2/HO-1/GPX4 pathway to protect against neurotoxicity in Wilson’s disease [107]. Conversely, in highly tumorigenic lung cancer cells (A549 CD133+), curcumin induces ferroptosis by suppressing the GSH-GPX4 and FSP1-CoQ10-NADH pathways, thereby inhibiting cancer cell self-renewal [108]. These findings highlight that the same compound may exhibit selective toxicity in different cell types, with its mechanisms of action and effects varying depending on the cell type.

#### 4.2.2. Flavonoid Inhibitors

Flavonoids, alongside polyphenolic compounds, constitute core components of plant-derived natural products and also exhibit ferroptosis-inhibitory potential. Myricitrin (Figure 9, compound **42**) demonstrated significant protective effects against cisplatin-induced injury in human renal tubular epithelial HK-2 cells. This protection may involve the inhibition of NOX4-mediated ferritinophagy, suggesting its potential as a candidate for cisplatin chemotherapy-induced acute kidney injury [109]. Amentoflavone (AMF, Figure 9, compound **43**), a natural biflavone compound possessing antioxidant, anti-inflammatory, and neuroprotective activities, exhibits significant neuroprotective effects against homocysteine-induced HT22 cell injury. This neuroprotection primarily involves the inhibition of ferroptosis-mediated inflammation, and activation of the SLC7A11/GPX4 axis is one of its potential underlying mechanisms [110].

#### 4.2.3. Hinokitiol

The α-hydroxyl ketone scaffold, widely distributed in natural molecules, possesses the ability to chelate transition metals. Hinokitiol (Hino, Figure 9, compound **44**), a member of the tropolone family, is a potent ferroptosis inhibitor containing an α-hydroxyl ketone scaffold. It effectively inhibits ferroptosis induced by the neurotoxin 6-hydroxydopamine (6-OHDA) in PC12 cells. Its mechanisms include stronger iron chelation compared to deferoxamine (DFO) and activation of the Nrf2/ARE signaling pathway, leading to the upregulation of antioxidant gene expression. In a zebrafish model, **44** exhibited protective effects against locomotor deficits and neuronal developmental abnormalities caused by 6-OHDA. In a mouse model, **44** demonstrated favorable BBB penetration. Furthermore, hinokitiol alleviates paclitaxel-induced neurotoxicity without compromising paclitaxel’s antitumor efficacy [111].

#### 4.2.4. Berberine

Berberine (BBR, Figure 9, compound **45**), an isoquinoline alkaloid extracted from *Coptis chinensis* Franch. and *Phellodendron chinense* Schneid, alleviates atherosclerosis by inhibiting the key driver ACSL4 [112]. In human umbilical vein endothelial cells (HUVECs) treated with erastin, BBR dose-dependently reversed the upregulation of ACSL4 and COX2, counteracted the downregulation of GPX4, SLC7A11, and FTH1, and suppressed the accumulation of ROS and LPO. Notably, ACSL4 overexpression completely abolished the protective effects of BBR, confirming the specificity of this mechanism. Animal experiments further demonstrated that a high dose of BBR (50 mg/kg) significantly improved vascular function in high-fat diet-fed ApoE^−/−^ mice, evidenced by reduced pulse wave velocity (PWV) and carotid artery intima-media thickness (IMT), decreased plaque area, and lowered levels of the aortic lipid peroxidation marker 4-HNE. More importantly, in a mouse model featuring vascular endothelium-specific overexpression of ACSL4, BBR still reversed atherosclerotic pathological changes without affecting blood lipid levels, clearly indicating that its action would be independent of lipid metabolism regulation [112]

#### 4.2.5. 7-Dehydrocholesterol (7-DHC)

Freitas’ team [113] and Li’s team [114] concurrently identified 7-dehydrocholesterol (7-DHC, Figure 9, compound **46**) as a potent natural inhibitor of ferroptosis. 7-DHC is an intermediate in cholesterol biosynthesis, synthesized by sterol-C5-desaturase (SC5D) and metabolized by 7-dehydrocholesterol reductase (DHCR7). These studies revealed that DHCR7 promotes ferroptosis, indicating that its catalytic substrate, 7-DHC, is a key factor in inhibiting ferroptosis. Both research groups independently investigated the mechanism of action of 7-DHC, with results converging on the ability of the conjugated diene system within ring B of the sterol backbone of 7-DHC to scavenge free radicals, thereby reducing lipid peroxidation. Li’s team [114]further demonstrated that pharmacological inhibition of DHCR7 led to the accumulation of 7-DHC in vivo, effectively treating renal ischemia-reperfusion injury in mice. Targeting key enzymes in cholesterol biosynthesis to modulate 7-DHC levels may represent a novel therapeutic strategy for clinical management of tumors or organ damage.

**Figure 10:**
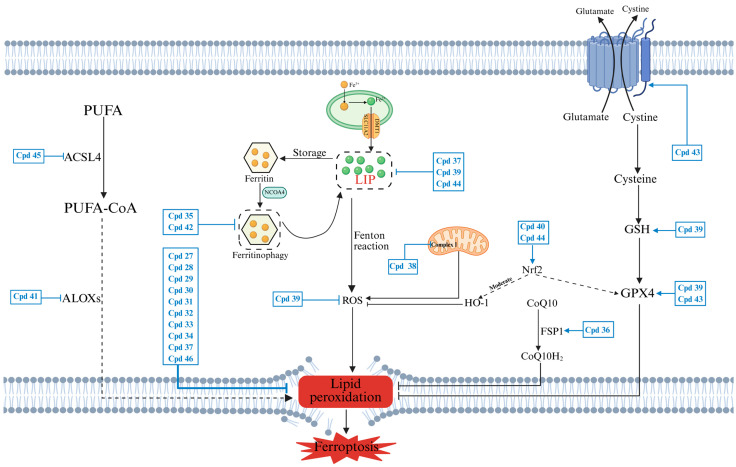
Schematic illustration of the modulatory mechanism of ferroptosis inhibitors. The diagram summarizes how the discussed small-molecule inhibitors regulate relevant ferroptosis pathways. Compound labels (e.g., Cpd 27) correspond to the numbers assigned in the text, Figure 8 and Figure 9 and Table 2. Created in Biorender. Chen, J. (2025) https://BioRender.com/bjnygmo (accessed on 16 November 2025).

Through in-depth exploration and structural optimization, numerous ferroptosis inhibitors with significantly enhanced activity, stability, and selectivity have been reported, laying a solid foundation for their applications. To facilitate the review and understanding of the modulatory mechanisms of these inhibitors, we referred to the summary form at the end of Section 3 and also summarized the ferroptosis inhibitors (Figure 10 and Table 2).

## 5. Discussion and Future Perspectives

Ferroptosis, characterized by iron-dependent lipid peroxidation, is a distinct form of regulated cell death different from apoptosis and necrosis, attracting significant attention. This discovery has expanded our knowledge of cell death pathways and potential therapeutic strategies for diseases involving ferroptosis dysregulation. However, challenges persist in understanding the molecular mechanisms of ferroptosis, particularly the intricate signaling pathways that drive its execution. The connections between ferroptosis and other forms of cell death, such as apoptosis and necroptosis, remain unclear. While lipid peroxidation is a hallmark event of ferroptosis, it also participates in other death pathways, making it difficult to precisely delineate the dominant role of ferroptosis and its interactions with other death mechanisms in complex disease pathologies, such as specific cancer stages or neuronal injury.

Addressing these challenges, future research should focus on several key directions. A fundamental priority is the in-depth dissection of molecular mechanisms of ferroptosis, which will lay the groundwork for developing more precise intervention tools. Understanding the interplay of ferroptosis with other cell death pathways in disease progression is crucial. Investigating the dominance of apoptosis in early stages versus ferroptosis later on, and how their coexistence impacts disease trajectory, is essential for identifying therapeutic windows. Concurrently, optimizing novel compounds to enhance selectivity, targeting efficiency, pharmacokinetics, and toxicity is necessary for clinical translation. Efforts should also actively explore traditional Chinese medicine for potential ferroptosis modulators and understand their mechanisms of action. Additionally, exploring the combination of classical compounds with novel agents, or designing structural combinations to achieve synergistic effects, represents another important strategy for improving therapeutic efficacy. Breakthroughs in these research directions will provide a solid foundation for developing innovative therapies that regulate ferroptosis, particularly for malignancies and diseases related to tissue injury.

## Figures and Tables

**Table 1:** Summary and comparison of ferroptosis inducers.

Classes	Compound Name (Number)	Mechanisms of Action	Related Disease	Refs.
Targeting the system x_c_^−^/GSH/GPX4 axis	Erastin (**1**)	Inhibit the activity of system x_c_^−^ to affect the synthesis of GSH; bind to VDAC2/3 to induce mitochondrial dysfunction	Colorectal cancer	[43,44,45]
	FA16 (**2**)	Inhibit the activity of system x_c_^−^	Human fibrosarcoma and hepatocellular carcinoma	[46]
	Sorafenib derivative (**3**)	Inhibit the activity of system x_c_^−^	Lung cancer	[49]
	Lepadin H (**4**)	Downregulate SLC7A11 and GPX4 expression and upregulate p53 and ACSL4 expression	Human cervical cancer and melanoma	[51]
	RSL3 (**5**) & RSL24 (**6**)	Covalently bind to GPX4 at the selenocysteine site	Diffuse large B-cell lymphoma and renal carcinoma	[52,53,54,55,87]
	C18 (**7**)	Covalently bind to GPX4 and form hydrogen bonds with other amino acid residues	Triple-negative breast cancer	[56]
	R-9i (**8**)	Covalently bind to GPX4 and form hydrogen bonds with other amino acid residues	Human fibrosarcoma	[58]
	Indirubin derivative (**9**)	Covalently bind to GPX4 and promote its ubiquitination	Colon cancer	[59]
	Tubastatin A (**10**)	Inhibit the activity of GPX4	Triple-negative breast cancer	[60]
	N6F11 (**11**)	Trigger the ubiquitination-mediated degradation of GPX4	Pancreatic cancer	[61]
	GIC-20 (**12**)	Dual induction of ferroptosis and apoptosis	Human fibrosarcoma	[62]
	JQ1 derivative (**13**)	Dual induction of ferroptosis and apoptosis	Triple-negative breast cancer	[66]
	AVB derivative (**14**)	Dual induction of ferroptosis and apoptosis	Triple-negative breast cancer	[67]
Targeting Fe^2+^ and ROS	CAPE derivative (**15**)	Excessively activate the HO-1 pathway to increase intracellular labile Fe^2+^ and ROS	Triple-negative breast cancer	[72]
	Pacidusin B (**16**)	Excessively activate the HO-1 pathway and inhibit the activity of system x_c_^−^	Human fibrosarcoma	[73]
	Fe(hino)_3_ (**17**)	Act as a redox-active complex to induce the Fenton reaction and deplete GSH	Triple-negative breast cancer	[74]
	Urea derivative (**18**)	Elevate ROS to trigger ferroptosis and autophagy	Colon cancer	[75]
	Benzenesulfo-namides derivative (**19**)	Dual induction of ferroptosis and apoptosis	Triple-negative breast cancer	[77]
	Sinomenine derivative (**20**)	Trigger ferritinophagy and increase intracellular labile Fe^2+^	Colorectal cancer	[78]
Targeting lipid metabolism	Seco-lupane triterpene derivative (**21**)	Upregulate the expression of ACSL4 and downregulate the expression of GPX4	Hepatocellular carcinoma	[79]
	(20 S)-Protopanaxatriol (**22**)	Upregulate ACSL4 transcription	Osteosarcoma	[80]
	Solanine (**23**)	Upregulate the expression of ALOX12B and ALOX5	Colorectal cancer	[81]
Targeting GPX4-independent antioxidant pathways	FSEN1 (**24**)	Non-competitively inhibit FSP1	Lung cancer	[82,83]
	BRQ derivative (**25**)	Inhibit DHODH expression to impair mitochondrial function and alleviate immunosuppression	Melanoma	[84]
	OART (**26**)	Inhibit the antioxidant pathways of DHODH and GPX4	Breast cancer	[86]

**Table 2:** Summary and comparison of ferroptosis inhibitors.

Source	Structure Classes	Compound Name (Number)	Mechanisms of Action	Related Disease/Model	Refs.
Synthetic inhibitors	Arylamines	Fer-1 (**27**)	Capture free radicals and inhibit lipid peroxidation	Ferroptosis model of HT-1080 cells induced by erastin/RSL3	[1]
		UAMC-4821 (**28**)	Capture free radicals and inhibit lipid peroxidation	Ferroptosis model of HT-1080 cells induced by ML162	[91]
	N-heterocyclic	Thienobenzodiazepine derivative (**29**)	Capture free radicals and inhibit lipid peroxidation	Ferroptosis model of HT22 cells induced by RSL3	[92]
		HW-3 derivative (**30**)	Capture free radicals and inhibit lipid peroxidation	Ferroptosis model of HT-1080 cells induced by RSL3	[93]
		NY-26 (**31**)	Capture free radicals and inhibit lipid peroxidation	Acute liver injury	[94]
		Phenyltetrazolium derivative (**32**)	Capture free radicals and inhibit lipid peroxidation	Ischemic stroke	[95]
	Phenothiazines and Phenazines	Phenothiazine derivative (**33**)	Capture free radicals and inhibit lipid peroxidation	Cardiomyopathy induced by doxorubicin	[96]
		Sulfonamide phenothiazine (**34**)	Capture free radicals and inhibit lipid peroxidation	Spinal cord injury	[97]
		Phenazine derivative (**35**)	Inhibit ferritinophagy and reduce liable Fe^2+^	Drug-induced liver injury	[98]
	Diphenylbutenyl	Diphenylbutenyl derivative (**36**)	Activate the FSP1-CoQ10 pathway	Ischemic stroke	[99]
	Hybrid	3-hydroxypyridin-4(1H)-one derivative (**37**)	Chelate intracellular Fe^2+^ and scavenge free radicals	Acute kidney injury	[100]
		UW-MD-190 (**38**)	Inhibit the activity of mitochondrial complex I and reduce mitochondrial respiration	Ferroptosis model of HT22 cells induced by RSL3/erastin/glutamate	[102]
Natural inhibitors	Polyphenolic	Moracin N (**39**)	Inhibiting GSH depletion, prevent GPX4 inactivation, reduce ROS and Fe^2+^ accumulation	Ferroptosis model of HT22 cells induced by erastin	[103]
		Thonningianin A (**40**)	Activate Keap1/Nrf2/HO-1 and AMPK/Nrf2/GPX4 pathways	Parkinson’s disease and Alzheimer’s disease	[104,105]
		Chicoric acid (**41**)	Inhibit the activity of ALOX15 and inhibit lipid peroxidation	Asthma	[106]
	Flavonoids	Myricitrin (**42**)	Inhibit ferritinophagy	Acute kidney injury	[109]
		Amentoflavone (**43**)	Activate the system x_c_^−^/GPX4 axis	Nerve injury induced by homocysteine	[110]
	______	Hinokitiol (**44**)	Chelate intracellular Fe^2+^ and activate the Nrf2/ARE signaling pathway	Parkinson’s disease	[111]
	______	Berberine (**45**)	Inhibit the activity of ACSL4 and inhibit lipid peroxidation	Atherosclerosis	[112]
	______	7-DHC (**46**)	Scavenge free radicals and inhibit lipid peroxidation	Renal ischemia-reperfusion injury	[113,114]

## Data Availability

No new data were created or analyzed in this study.
